# Association of ATM and BMI‐1 genetic variation with breast cancer risk in Han Chinese

**DOI:** 10.1111/jcmm.13650

**Published:** 2018-04-24

**Authors:** Li‐Ling Yue, Fu‐Chao Wang, Ming‐Long Zhang, Dan Liu, Ping Chen, Qing‐Bu Mei, Peng‐Hui Li, Hong‐Ming Pan, Li‐Hong Zheng

**Affiliations:** ^1^ Department of Biogenetics Qiqihar Medical University Qiqihar Heilongjiang China; ^2^ Clinical Laboratory Daqing Oilfield General Hospital Daqing Heilongjiang China; ^3^ Department of Biochemistry Qiqihar Medical University Qiqihar Heilongjiang China

**Keywords:** association, ATM gene, BMI‐1 gene, breast cancer, case–control, risk

## Abstract

We tested the hypothesis that genetic variation in ATM and BMI‐1 genes can alter the risk of breast cancer through genotyping 6 variants among 524 breast cancer cases and 518 cancer‐free controls of Han nationality. This was an observational, hospital‐based, case–control association study. Analyses of single variant, linkage, haplotype, interaction and nomogram were performed. Risk was expressed as odds ratio (OR) and 95% confidence interval (CI). All studied variants were in the Hardy‐Weinberg equilibrium and were not linked. The mutant allele frequencies of rs1890637, rs3092856 and rs1801516 in ATM gene were significantly higher in cases than in controls (*P* = .005, <.001 and .001, respectively). Two variants, rs1042059 and rs201024480, in BMI‐1 gene were low penetrant, with no detectable significance. After adjustment, rs189037 and rs1801516 were significantly associated with breast cancer under the additive model (OR: 1.37 and 1.52, 95% CI: 1.10‐1.71 and 1.14‐2.04, *P*: .005 and .005, respectively). In haplotype analysis, haplotypes A‐C‐G‐G (in order of rs189037, rs3092856, rs1801516 and rs373759) and A‐C‐A‐A in ATM gene were significantly associated with 1.98‐fold and 6.04‐fold increased risk of breast cancer (95% CI: 1.36‐2.90 and 1.65‐22.08, respectively). Nomogram analysis estimated that the cumulative proportion of 3 significant variants in ATM gene was about 12.5%. Our findings collectively indicated that ATM gene was a candidate gene in susceptibility to breast cancer in Han Chinese.

## 1 INTRODUCTION

Breast cancer is a multifactorial disease.^1^ Growing evidence supports a polygenic basis of breast cancer.^2^ Family history was well‐known as an attributable risk factor for breast cancer.^3^ Dozens of breast cancer‐susceptibility genes have been identified through analysing genome‐scale data of various nationalities and races.^4^, ^5^, ^6^ However, only a tiny proportion of genetic variants have been consistently validated by some but not all genome‐scale analyses.^7^, ^8^ Initial findings from genome‐scale analyses seem promising. However, sequent efforts are needed to establish a catalogue of ethnicity‐specific common variation in the genome.

More and more geneticists are beginning to appreciate the role of candidate gene approach.^9^, ^10^ In general, candidate gene refers to the gene with known biological function in directly or indirectly regulating the investigated traits.^11^ Candidate gene approach has been widely applied to genetic association studies.^12^, ^13^ Using this approach, we genotyped 2 candidate genes, HMGB1 and RAGE, and found a cumulative impact of multiple variants on breast cancer risk.^14^ In this study, we focused on 2 cancer‐predisposing genes, ATM and BMI‐1, in association with breast cancer. The involvement of ATM and BMI‐1 in carcinogenesis is biologically plausible.^15^, ^16^, ^17^, ^18^ ATM is an acronym for ataxia telangiectasia mutated. ATM is a serine/threonine kinase involving in DNA damage repair, cell arrest and apoptosis, and chromatin remodelling.^19^, ^20^ Genetic defects in ATM can cause multiple system dysfunctions and increase tumour susceptibility.^21^, ^22^, ^23^, ^24^ BMI‐1 is an acronym for B‐cell‐specific Moloney murine leukaemia virus integration site 1. BMI‐1 is a polycomb protein that plays an important role in tumour cell development and maintaining stem cell populations of many cell lineages.^25^ We therefore develop a hypothesis that ATM and BMI‐1 genes are candidate genes of breast cancer. To test this hypothesis, we genotyped 4 variants in ATM gene and 2 variants in BMI‐1 gene in 524 breast cancer cases and 518 cancer‐free controls from Heilongjiang province, China to see whether they can alter the risk of breast cancer.

## 2 METHODS

### 2.1 Study design

This is an observational, hospital‐based, case–control, genetic association study, as previously reported.^14^

### 2.2 Study participants

Only female participants who were Han Chinese were recruited in this study. Recruitment was conducted in 4 hospitals of Heilongjiang province, China, that is, Daqing Oilfield General Hospital, The 2nd and 3rd Affiliated Hospitals of Qiqihar Medical University and Qiqihar Jianhua Hospital. This study was conducted during the period from January 2013 to August 2015.

### 2.3 Ethical approval

The conduct of this study was approved by the Ethics Committee of Qiqihar Medical University. Written informed consent was obtained from all study participants at the time of recruitment. This study complied with the Declaration of Helsinki.

### 2.4 Breast cancer diagnosis

A patient was recorded to have breast cancer if he or she had either newly diagnosed or histopathologically confirmed or previously untreated breast cancer.

### 2.5 Eligibility criteria

Control participants had no clinical evidence of any types of cancer except non‐melanoma skin cancer. All controls reported to have no family history of cancer. No restriction was placed on age and tumour stage at the time of recruitment.

### 2.6 Sample size

In total, 1042 female participants were eligible for inclusion. There were 524 cases with breast cancer and 518 controls free of cancer.

### 2.7 Information collection

The following information was collected from cases with breast cancer, including age of first onset, age of menarche, menopausal age, family history of cancer, invasion depth (T1‐T4), tumour stage (I‐III) and lymph node. From controls free of cancer, age at enrolment and age of menarche were recorded.

### 2.8 Phlebotomy

Venous blood samples (3 mL) were drawn into 5‐mL vacutainer tubes containing K_3_‐EDTA tubes from each participant. Plasma was separated by centrifugation at 4°C and kept frozen in a deep freezer at −80°C until assayed.

### 2.9 DNA extraction

Genomic DNA was extracted from leucocytes using the phenol‐cholesterol method according to a standard procedure.

### 2.10 Variant selection

Four variants in ATM gene were selected, including rs189037 (A‐111G), rs3092856 (1380His>Tyr or C4138T), rs1801516 (1853Asp>Asn) and rs373759 (126713G>A). Two variants were selected from BMI‐1 gene, including rs1042059 (18Cys>Tyr) and rs201024480 (310Ser>Asn). The selection of these variants was based on published papers 25, 26, 27, 28, 29, 30 and the NCBI‐Gene website analysis (https://www.ncbi.nlm.nih.gov/gene/). In this study, no restriction was placed on the cut‐off point of minor allele frequency of selected variants, as available linkage analyses only identified some rare variants of genes involved in DNA repair, including ATM gene under investigation, and these variants were associated with a moderate risk of breast cancer.^1^, ^31^

### 2.11 Genotyping

Genomic sequences of 6 variants in ATM and BMI‐1 genes were amplified by polymerase chain reaction. Genotyping was determined using ligase detection reaction method.^32^ For each allele of a single variant, a specific probe was synthesized, and an additional common probe capped with 6‐carboxy‐fluorescein at the 3′ end and with horylated at the 5′ end was also synthesized. The primers and probes are available upon reasonable request.

Using the same method, 60 randomly selected samples were re‐genotyped, and the results were completely identical.

Genotyping was performed by laboratory workers in a manner blind to the case–control status and related characteristics of study participants.

### 2.12 Statistical analyses

Continuous variables expressed as mean (standard deviation) were compared between cases and controls using *t* test or Mann‐Whitney *U* test depending on its distribution. Categorical variable expressed as percentage were compared using the χ^2^ test. Testing Hardy‐Weinberg equilibrium in control participants, as well as genotype and allele differences between cases and controls was completed by the χ^2^ test or Fisher's exact test. Breast cancer risk conferred by genetic variants was calculated using logistic regression analysis after adjusting for age and age of menarche. Effect size was indexed as odds ratio (OR) and 95% confidence interval (95% CI). In addition, given the limited sample size of this study, an internal validation was performed by evenly and randomly splitting all study participants into 2 groups, *viz*. training group (n = 512) and testing group (n = 512). Risk prediction for breast cancer was, respectively, conducted in both groups.

Linkage analysis was performed using the HaploView software Release 4.2 available at https://www.broadinstitute.org/haploview/haploview, and linkage magnitude was expressed as D prime (D’).

Haplotype analysis was performed using the HAPLO.STATS program after controlling for age and age of menarche. This program was realized using the R Project for Statistical Computing Release 3.4.3 available at https://www.r-project.org/.

Interaction analysis was performed using the open‐source multifactor dimensionality reduction (MDR) software Release 3.0.2 available at http://www.multifactordimensionalityreduction.org/.

A risk nomogram calculator was produced based on baseline information and significant variants identified. The nomogram was depicted using regression modelling strategies (rms) program. This program also was realized using the R Project for Statistical Computing Release 3.4.3. Specifically, a nomogram is a 2‐dimensional diagram that allows the approximate graphical computation of a mathematical function, and its accuracy can be justified by the concordance index (C‐index).^33^ The C‐index measures the magnitude of concordance between predicted probabilities and the actual chance of having breast cancer.

Unless otherwise indicated, analyses were performed with the STATA software Release 13.0 (StataCorp LP, College Station, TX, USA).

## 3 RESULTS

### 3.1 Baseline characteristics

This study involved 524 cases with breast cancer and 518 controls free of cancer. Table 1 shows the comparison of baseline characteristics between cases and controls. Mean age and mean age of menarche differed significantly between the 2 groups (both *P* < .001). In cases, mean menopausal age was 50.19 years and only a small proportion of cases had a family history of cancer (5.95%). In case of invasion depth, 49.69%, 42.77%, 3.77% and 3.77% of cases were in T1, T2, T3 and T4, respectively. Tumour stages II (49.27%) and III (46.10%) accounted for the majority of breast cancer cases, and 42.47% of cases had positive lymph nodes.

**Table 1:** The baseline characteristics of study participants

Characteristics	Cases	Controls	*P* value
Sample size	524	518	
Age (y)	53.76 (12.62)	56.49 (10.04)	<.001
Age of menarche (y)	14.61 (1.65)	13.04 (1.12)	<.001
Menopausal age (y)	50.19 (3.98)	n.a.	
Family history of cancer	5.95%	0.00%	<.001
Invasion depth (n = 477)		n.a.	
T1	49.69% (n = 237)		
T2	42.77% (n = 204)		
T3	3.77% (n = 18)		
T4	3.77% (n = 18)		
Tumour stage (n = 410)		n.a.	
I	4.63% (n = 19)		
II	49.27% (n = 202)		
III	46.10% (n = 189)		
Positive lymph node (n = 511)	42.47% (n = 217)	n.a.	

n.a.: not available. Data are expressed as mean (standard deviation) or percentage.

### 3.2 Hardy‐Weinberg equilibrium test

All studied variants were consistent with the distributions predicted by the Hardy‐Weinberg equilibrium in controls (all *P* > .01).

### 3.3 Linkage analysis

Linkage analysis indicated that the 4 variants in ATM gene and the 2 variants in BMI‐1 gene were not correlated (Figure S1).

### 3.4 Single variant analysis

Table 2 shows the genotype and allele distributions/frequencies of 6 variants in ATM and BMI‐1 gene. The mutant allele frequencies of rs1890637 (A), rs3092856 (T) and rs1801516 (A) were significantly higher in cases than in controls (*P* = .005, <.001 and .001, respectively). The genotype distributions differed remarkably significantly for rs3092856 and rs1801516 between cases and controls (both *P* < .001). The 2 variants, rs1042059 and rs201024480, in BMI‐1 gene belonged to low‐penetrance mutations, and there was no hint of significance with breast cancer risk. The genotype and allele comparisons of 6 studied variants between breast cancer cases, respectively, stratified by tumour stage, invasion depth, lymph node and controls are presented in Table S1.

**Table 2:** Genotype and allele distributions of 6 studied variants between cases and controls, and genotype‐based risk prediction for breast cancer

Gene: Variant	Cases (n = 524)	Controls (n = 518)	*P* value	OR, 95% CI, *P* value*
ATM: rs189037				
GG	166 (31.68%)	196 (37.84%)	.012	Reference group
GA	262 (50.00%)	258 (49.81%)		1.18, 0.84‐1.64, .341
AA	96 (18.32%)	64 (12.36%)		2.02, 1.28‐3.18, .002
A	43.32%	37.26%	.005	
ATM: rs3092856				
CC	445 (84.92%)	474 (91.51%)	<.001	Reference group
CT	67 (12.79%)	42 (8.11%)		1.36, 0.82‐2.24, .232
TT	12 (2.29%)	2 (0.39%)		4.95, 0.96‐25.52, .056
T	8.68%	4.44%	<.001	
ATM: rs1801516				
GG	351 (66.98%)	380 (73.36%)	<.001	Reference group
GA	146 (27.86%)	133 (25.68%)		1.22, 0.86‐1.72, .262
AA	27 (5.15%)	5 (0.97%)		8.06, 2.41‐26.92, .001
A	19.08%	14.15%	.001	
ATM: rs373759				
GG	161 (30.37%)	162 (31.27%)	.080	Reference group
GA	247 (47.14%)	269 (51.93%)		0.89, 0.63‐1.26, .520
AA	116 (22.14%)	87 (16.80%)		1.10, 0.72‐1.70, .653
A	45.71%	42.76%	.176	
BMI‐1: rs1042059				
GG	511 (97.52%)	506 (97.68%)	.862	Reference group
GA	13 (2.48%)	12 (2.32%)		0.94, 0.36‐2.42, .892
AA	0 (0.0%)	0 (0.0%)		Unavailable
A	1.24%	1.16%	.863	
BMI‐1: rs201024480				
AA	515 (98.28%)	511 (98.65%)	1.000	Reference group
AG	8 (1.53%)	7 (1.35%)		1.03, 0.27‐3.97, .965
GG	1 (0.19%)	0 (0.0%)		Unavailable
G	0.95%	0.68%	.480	

OR, odds ratio; 95% CI, 95% confidence interval. *The *P* values were calculated after adjusting for age and age of menarche in a logistic regression analysis.

When the wild homozygous genotype was taken as the reference group, the mutant homozygous genotypes of rs189037 (AA) and rs1801516 (AA) were associated with the significant risk (OR: 2.02 and 8.06, 95% CI: 1.28‐3.18 and 2.41‐26.92, *P*: .002 and .001) of breast cancer after adjusting for age and age of menarche (Table 2), and this association was retained in both training and testing groups, as shown in Table S2. For all variants, the comparison of heterozygous genotype with wild homozygous genotype was non‐significant in all study participants, as well as in both training and testing groups (Table S2).

In the light of small number of mutant homozygotes for some variants, risk prediction for breast cancer was explored under additive and dominant models of inheritance (Table 3). After adjusting for age and age of menarche, rs189037 and rs1801516 were remarkably associated with breast cancer risk under the additive model (OR: 1.37 and 1.52, 95% CI: 1.10‐1.71 and 1.14‐2.04, *P*: .005 and .005, respectively), whereas this association was marginally significant for rs189037 in training group (OR: 1.42, 95% CI: 1.07‐1.99, *P*: .038) and for rs189037 and rs1801516 in testing group (OR: 1.33 and 1.60, 95% CI: 1.02‐1.72 and 1.05‐2.43, *P*: .041 and .027, respectively) (Table S3). In addition, the adjusted association of rs3092856 and rs1801516 with breast cancer was marginally significant, respectively, under the additive and dominant models (OR: 1.58 and 1.39, 95% CI: 1.04‐2.39 and 1.00‐1.93, *P*: .031 and .048), and significance persisted in both training and testing groups (Table S3).

**Table 3:** The unadjusted and adjusted risk prediction of 6 studied variants for breast cancer under additive and dominant models, respectively

Gene: Variant	Model*	Additive model	Dominant model
ATM: rs189037	Unadjusted	1.30, 1.09‐1.56, .004	1.32, 1.02‐1.70, .037
	Adjusted	1.37, 1.10‐1.71, .005	1.34, 0.98‐1.85, .067
ATM: rs3092856	Unadjusted	1.88, 1.33‐2.67, <.001	1.91, 1.29‐2.83, .001
	Adjusted	1.58, 1.04‐2.39, .031	1.55, 0.96‐2.49, .071
ATM: rs1801516	Unadjusted	1.46, 1.16‐1.84, .001	1.36, 1.04‐1.77, .026
	Adjusted	1.52, 1.14‐2.04, .005	1.39, 1.00‐1.93, .048
ATM: rs373759	Unadjusted	1.13, 0.95‐1.34, .175	1.03, 0.79‐1.33, .848
	Adjusted	1.03, 0.84‐1.28, .756	0.95, 0.69‐1.32, .758
BMI‐1: rs1042059	Unadjusted	1.07, 0.48‐2.37, .862	1.07, 0.48‐2.37, .862
	Adjusted	0.94, 0.36‐2.42, .892	0.94, 0.36‐2.42, .892
BMI‐1: rs201024480	Unadjusted	1.37, 0.54‐3.45, .505	1.28, 0.47‐3.45, .632
	Adjusted	1.03, 0.27‐3.97, .965	1.03, 0.27‐3.97, .965

Data are expressed as odds ratio, 95% confidence interval, *P* value. *The *P* values were calculated after adjusting for age and age of menarche in a logistic regression analysis.

### 3.5 Haplotype analysis

Haplotype analysis was, respectively, conducted in ATM gene and BMI‐1 gene, as they are mapped on different chromosomes. The derived haplotype frequencies and risk estimates for breast cancer are presented in Table 4. In ATM gene, haplotype G‐C‐G‐G (alleles arranged by order of rs189037, rs3092856, rs1801516 and rs373759, with the same hereafter) was the most common, and its frequency was significantly higher in controls than in cases (30.82% vs 20.74%, *P* < .001). A low‐penetrance haplotype A‐C‐A‐A was overrepresented in cases than in controls (3.98% vs 0.95%, *P* = .001).

**Table 4:** Haplotype frequencies of variants in ATM and BMI1 genes between cases and controls, and haplotype‐based risk prediction for breast cancer

Haplotype^a^	Hap‐Score	All, %	Cases (n = 524), %	Controls (n = 518), %	Sim *P* value	OR	95% lower limit	95% upper limit
ATM gene								
G‐C‐G‐G	−4.77	25.78	20.74	30.82	<.001	1.00	Reference group	
G‐C‐G‐A	−1.06	20.69	20.88	19.99	.287	1.55	0.98	2.12
A‐C‐G‐G	1.32	17.96	20.25	14.90	.019	1.98	1.36	2.90
A‐C‐G‐A	−0.21	13.83	12.55	16.29	.838	1.22	0.87	1.70
G‐C‐A‐G	−0.34	5.23	5.13	5.69	.736	1.45	0.80	2.65
G‐C‐A‐A	1.56	3.65	3.99	3.26	.116	1.76	0.92	3.40
A‐C‐A‐G	1.64	3.60	3.79	3.67	.102	1.54	0.81	2.93
A‐C‐A‐A	3.41	2.69	3.98	0.95	.001	6.04	1.65	22.08
G‐T‐G‐A	2.03	2.01	2.52	1.79	.043	2.39	0.88	6.45
G‐T‐G‐G	1.85	1.42	1.90	0.95	.065	1.79	0.45	7.15
A‐T‐G‐A	1.67	1.00	1.20	0.30	.093	2.19	0.50	9.58
BMI‐1 gene								
G‐A	−0.58	97.98	97.81	98.17	0.559	1.00	Reference group	
A‐A	0.17	1.20	1.24	1.16	0.842	1.08	0.49	2.39

Hap‐Score, haplotype score, Sim *P* value, simulated *P* value; OR, odds ratio.

a In ATM gene, alleles are arranged according to the order of rs189037, rs3092856, rs1801516 and rs373759, and in BMI‐1 gene, alleles are arranged according to the order of rs1042059 and rs201024480.

When taking the most common haplotype (G‐C‐G‐G) as the reference group, haplotypes A‐C‐G‐G and A‐C‐A‐A in ATM gene were significantly associated with 1.98‐fold and 6.04‐fold increased risk of breast cancer (95% CI: 1.36‐2.90 and 1.65‐22.08, respectively). Other haplotypes carried no significance in association with breast cancer.

### 3.6 Interaction analysis

The interaction of 6 studied variants is illustrated in Figure S2. The contribution of rs1801516 to breast cancer risk was the largest (1.27%), followed by rs3092856 (1.01%). This figure highlights the amount of information gained about the case–control status by any 2 variants. A red or orange line denotes a positive information gain and can be explained as a synergistic or non‐additive relation. By contrast, a blue or green line denotes a loss of information and can be explained as redundancy or linkage disequilibrium. The 2 rare variants in BMI‐1 gene were most likely interacted with rs189037 and rs373759 in the ATM gene. The relationship between the 4 variants in ATM gene was redundant in view of their weak linkage profile.

### 3.7 Nomogram presentation

**Figure 1:**
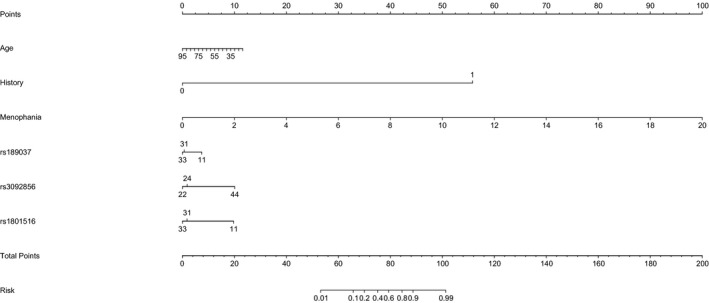
Nomogram calculator in prediction for the risk of breast cancer. The magnitude of each factor in prediction for the risk of breast cancer can be estimated through drawing a vertical line from the measured value of each factor to the horizontal “Points” line, and each point is recorded. The total point is calculated by summing up all individual points and is marked on the horizontal “Total Points” line. Then, a vertical line starting from this total point is projected on the horizontal “Risk” line, and the value denotes the risk of having breast cancer

A nomogram calculator is presented in Figure 1, by including age, family history, age of menarche as well as 3 significant variants (rs189037, rs3092856, rs1801516). The magnitude of age of menarche was the strongest, following by family history, age, rs3092856, rs1801516 and rs189037. The cumulative contributory proportion of 3 significant variants to breast cancer was estimated to be 12.5%. The nomogram was somewhat good in prediction accuracy, with the C‐index estimated to be 0.72 (*P* = .011).

## 4 DISCUSSION

Our findings partly verified the hypothesis of this study, by showing that ATM gene might be a candidate gene of breast cancer in Han Chinese. In particular, we have identified 2 variants in ATM gene, rs189037 and rs1801516, which may play a dominant role, as well as a possible joint role, in susceptibility to breast cancer risk. Despite no detectable significance, this study represents the first attempt to study the association between BMI‐1 genetic variation and breast cancer.

The association of ATM genetic variation with breast cancer has been widely evaluated in Western countries,^34^, ^35^, ^36^, ^37^, ^38^, ^39^ whereas it was rarely reported in China. A recent meta‐analysis on different risk measures of ATM‐breast cancer relation documented that the cumulative risk of breast cancer attributable to ATM genetic variants was 6.02% by 50 years of age, and it reached 32.83% by 80 years of age,^40^ leading to the conjecture that ATM might be involved in the pathogenesis of breast cancer. In fact, our nomogram analysis showed that the cumulative contributory proportion of 3 significant variants in ATM gene was about 12.5%, falling in between the proportions mentioned above. The mean age of our cases with breast cancer was 53.76 years, prompting us to speculate that the magnitude of ATM gene in predisposition to breast cancer was relatively strong in Chinese.

Two variants in ATM gene were under wide investigations. A meta‐analysis of 9 studies indicated that ATM gene rs1801516 might not be a breast cancer‐susceptibility locus in populations of European and Amerindian origins.^41^ By contrast in this study, this variant differed significantly in both allele and genotype frequencies in Han Chinese. In particular, the mutant AA genotype of rs1801516 was significantly associated with an 8‐fold increase in susceptibility to breast cancer. The reasons are manifold for this divergence. A rational explanation lies in genetic diversity across races. For instance, a given susceptibility gene may contribute to the pathogenesis of breast cancer in one population but not in another. In addition, chance, bias and confounding infiltrated in observational case–control studies can often cause an artificial association, especially for small‐size studies. Another variant, rs189037, in the promoter region of ATM gene was found to be a risk factor for breast cancer,^42^ which was in agreement with the findings of the present study. The mutation A allele of rs189037 was overrepresented in breast cancer cases relative to controls, and carriers of the AA genotype were roughly 2 times more likely to develop breast cancer. Considering the increased risk conferred by a single allele may be small, we further explored the joint impact of ATM genetic variants on breast cancer susceptibility.

In further haplotype analysis, we observed that that haplotype A‐C‐A‐A carrying the mutations of both rs189037 (the 1st allele) and rs1801516 (the 3rd allele) was overrepresented in cases relative to controls, and its association with breast cancer was statistically significant. This observation not only reinforced the results of our single variant analysis, but also suggested the joint contribution of different loci in ATM gene on the development of breast cancer. However, our interaction analysis of 6 studied variants in both ATM and BMI‐1 gene failed to reveal any synergistic effect. It is possible that the variants under study may be in linkage with other causative mutations in ATM gene. We agree that additional confirmation of our findings is necessary to unravel the genetic involvement of ATM gene in breast carcinogenesis.

Besides the candidate ATM gene, we have assessed the association between BMI‐1 gene 2 variants and breast cancer risk. This is, to the best of our knowledge, the first attempt in the literature that has tested this association. However, as the 2 variants are low penetrant in nature, we did not detect any significant association with breast cancer in both single variant and haplotype analyses. So, we proposed that BMI‐1 gene might not be a breast cancer‐susceptibility candidate.

Several limitations should be considered in this study. First, this is an observational case–control association study, and our data cannot be used to prove the cause–effect relationship between ATM gene and breast cancer risk. Second, the coverage of genetic variation in ATM and BMI‐1 genes was limited. Third, all participants were collected from 4 local hospitals, and multi‐centre confounding cannot be ruled out. Fourth, this study involved only Chinese women of Han nationality, and extrapolation of our results to the other nationalities was only speculative.

Taken together, our findings indicated that ATM gene was a candidate gene in susceptibility to breast cancer in Han Chinese. This study suggests genetic heterogeneity in diverse racial groups and underscores the necessity for race‐specific variation screening. In addition, our findings might be useful for the identification of high‐risk youth women by genotyping ATM genetic variants before the cumulative exposure of obvious risk factors, and meanwhile this study offers some helpful hints in understanding the pathophysiology of breast cancer and other solid tumours.

## CONFLICTS OF INTEREST

None declared.

## AUTHOR CONTRIBUTIONS

L.H.Z and H.M.P planned and designed the study, and directed its implementation; L.L.Y and F.H.W drafted the protocol; F.H.W and PC obtained statutory and ethics approvals; M.L.Z, PC and DL contributed to data acquisition; DL, P.H.L and Q.B.M. had access to all raw data; M.L.Z and Q.B.M did the data preparation, quality control and data analysis; L.H.Z and L.L.Y wrote the manuscript.
