# Supplementary material for: Association of ATM and BMI‐1 genetic variation with breast cancer risk in Han Chinese
Source: J Cell Mol Med. 2018 Apr 24;22(7):3671–8. doi: 10.1111/jcmm.13650 (PMC6010860; doi:10.1111/jcmm.13650)

Supplementary Table 1. The genotype and allele comparisons of six studied variants in ATM and BMI-1 genes between breast cancer cases respectively stratified by tumor stage, invasion depth, lymph node and controls.

Gene: Variant		Controls (n=518)	Cases						*p1*	*p2*	*p3*	*p4*	*p5*	*p6*
			Tumor stage		Invasion depth		Lymph node							
			Stage I-II (n=221)	Stage III (n=189)	T1 (n=237)	T2-T4 (n=240)	Positive (n=217)	Negative (n=294)						
ATM: rs189037	GG	196	70	55	84	65	59	103						
	GA	258	111	101	115	126	114	141	0.072	0.051	0.380	0.002	0.003	0.181
	AA	64	40	33	38	49	44	50						
	A	37.26%	43.21%	44.18%	40.30%	46.67%	46.54%	40.99%	0.032	0.018	0.260	0.001	0.001	0.138
ATM: rs3092856	CC	474	193	157	204	200	182	253						
	CT	42	21	27	29	32	29	36	0.007	0.001	0.031	<0.001	0.001	0.021
	TT	2	7	5	4	8	6	5						
	T	4.44%	7.92%	9.79%	7.81%	10.00%	9.45%	7.82%	0.007	<0.001	0.008	<0.001	<0.001	0.005
ATM: rs1801516	GG	380	151	128	166	153	138	206						
	GA	133	58	50	61	72	65	75	0.001	0.001	0.012	<0.001	<0.001	0.006
	AA	5	12	11	10	15	14	13						
	A	13.80%	18.55%	19.05%	17.09%	21.25%	21.43%	17.18%	0.020	0.015	0.096	<0.001	<0.001	0.067
ATM: rs373759	GG	162	74	56	78	67	65	92						
	GA	269	101	86	109	118	107	131	0.243	0.049	0.230	0.126	0.446	0.026
	AA	87	46	47	137	55	45	71						
	A	42.76%	43.67%	47.62%	59.10%	47.50%	45.39%	46.43%	0.748	0.013	<0.001	0.084	0.353	0.152
BMI-1: rs1042059	GG	506	216	184	230	234	211	287						
	GA	12	5	5	7	6	6	7	0.964	0.801	0.604	0.877	0.720	0.954
	AA	0	0	0	0	0	0	0						

	A	1.16%	1.13%	1.32%	1.48%	1.25%	1.38%	1.19%	0.964	0.802	0.606	0.878	0.721	0.954
BMI-1: rs201024480	AA	511	219	186	235	235	213	290						
	AG	7	2	3	2	4	3	4	1.000	0.732	0.727	0.366	0.416	1.000
	GG	0	0	0	0	1	1	0						
	G	0.68%	0.45%	0.79%	0.42%	1.25%	1.15%	0.68%	0.614	0.732	0.728	0.259	0.354	1.000

Data are expressed in count or percentage. The *p1*, *p2*, *p3*, *p4*, *p5* and *p6* respectively denote the comparisons of breast cancer cases with stage I-II, stage III, invasion depth T1, invasion depth T2-T4, positive lymph node and negatively lymph node with controls, and they were calculated using the χ^2 test or Fisher's exact test, where appropriate.

Supplementary Table 2. The genotype-based risk prediction of six studied variants in ATM and BMI-1 genes for breast cancer in both training and testing groups.

Gene: Variant	Genotype	Training group (n=521)	Testing group (n=521)
ATM: rs189037	GG	Reference group	Reference group
	GA	1.45, 0.91-2.31, 0.116	1.03, 0.67-1.60, 0.664
	AA	2.38, 1.29-3.79, 0.006	1.59, 1.19-3.50, 0.009
ATM: rs3092856	CC	Reference group	Reference group
	CT	1.27, 0.62-2.60, 0.517	1.50, 0.74-3.04, 0.257
	TT	5.47, 0.87-10.68, 0.266	2.16, 0.73-16.18, 0.301
ATM: rs1801516	GG	Reference group	Reference group
	GA	1.25, 0.78-2.02, 0.358	1.17, 0.71-1.92, 0.546
	AA	9.44, 2.32-17.51, 0.003	6.08, 1.04-11.42, 0.017
ATM: rs373759	GG	Reference group	Reference group
	GA	0.91, 0.57-1.46, 0.708	0.84, 0.50-1.40, 0.499
	AA	1.26, 0.71-2.37, 0.395	1.04, 0.64-1.80, 0.597
BMI-1: rs1042059	GG	Reference group	Reference group
	GA	1.03, 0.27-3.88, 0.970	0.87, 0.21-3.01, 0.866
	AA	Unavailable	Unavailable
BMI-1: rs201024480	AA	Reference group	Reference group
	AG	1.06, 0.18-6.08, 0.952	0.99, 0.33-13.38, 0.951
	GG	Unavailable	Unavailable

Data are expressed as odds ratio, 95% confidence interval, *p* value. The *p* values were calculated after adjusting for age and age of menarche in a logistic regression analysis.

Supplementary Table 3. The unadjusted and adjusted risk prediction of six studied variants in ATM and BMI-1 genes for breast cancer in both training and testing groups under additive and dominant models, respectively.

		Additive model		Dominant model	
**Gene: Variant**	**Model***	**Training group (n=521)**	**Testing group (n=521)**	**Training group (n=521)**	**Testing group (n=521)**
ATM: rs189037	Unadjusted	1.41, 1.10-1.81, 0.007	1.21, 1.02-1.58, 0.026	1.52, 1.06-2.19, 0.024	1.20, 0.84-1.73, 0.314
	Adjusted	1.42, 1.07-1.99, 0.038	1.33, 1.02-1.72, 0.041	1.45, 0.93-2.27, 0.104	1.04, 0.66-1.66, 0.861
ATM: rs3092856	Unadjusted	1.68, 1.03-2.74, 0.037	1.97, 1.24-3.04, 0.008	1.65, 1.05-2.78, 0.017	2.27, 1.26-4.12, 0.006
	Adjusted	1.92, 1.42-3.02, 0.004	1.26, 1.01-1.70, 0.038	1.49, 0.92-2.28, 0.096	1.58, 0.79-3.06, 0.197
ATM: rs1801516	Unadjusted	1.62, 1.15-2.28, 0.006	1.21, 0.96-1.79, 0.074	1.51, 1.03-2.21, 0.034	1.05, 0.72-1.53, 0.814
	Adjusted	1.41, 1.08-1.84, 0.001	1.60, 1.05-2.43, 0.027	1.28, 1.01-2.77, 0.048	1.53, 1.08-2.74, 0.037
ATM: rs373759	Unadjusted	1.06, 0.83-1.35, 0.659	1.26, 0.85-2.23, 0.759	0.91, 0.63-1.32, 0.623	1.10, 0.52-1.78, 0.320
	Adjusted	0.88, 0.61-1.20, 0.620	1.02, 0.80-1.57, 0.407	1.05, 0.48-1.38, 0.215	0.84, 0.52-1.37, 0.491
BMI-1: rs1042059	Unadjusted	0.95, 0.45-1.63, 0.530	1.26, 0.43-5.53, 0.262	1.05, 0.45-4.63, 0.730	1.12, 0.63-5.53, 0.562
	Adjusted	0.94, 0.40-2.07, 0.364	0.89, 0.37-2.83, 0.607	1.27, 0.35-4.64, 0.714	0.81, 0.21-3.21, 0.766
BMI-1: rs201024480	Unadjusted	1.76, 0.47-3.51, 0.726	1.08, 0.36-3.26, 0.885	1.34, 1.04-4.29, 0.035	1.21, 1.00-3.20, 0.051
	Adjusted	0.98, 0.15-3.43, 0.640	1.05, 0.12-9.39, 0.966	1.05, 0.18-6.00, 0.955	1.01, 0.43-13.38, 0.821

Data are expressed as odds ratio, 95% confidence interval, *p* value. The *p* values were calculated after adjusting for age and age of menarche in a logistic regression analysis.

Supplementary Figure 1. The linkage pattern of six studied variants in ATM and BMI-1 genes. Numbers in the diamond represent the D prime.

ATM gene

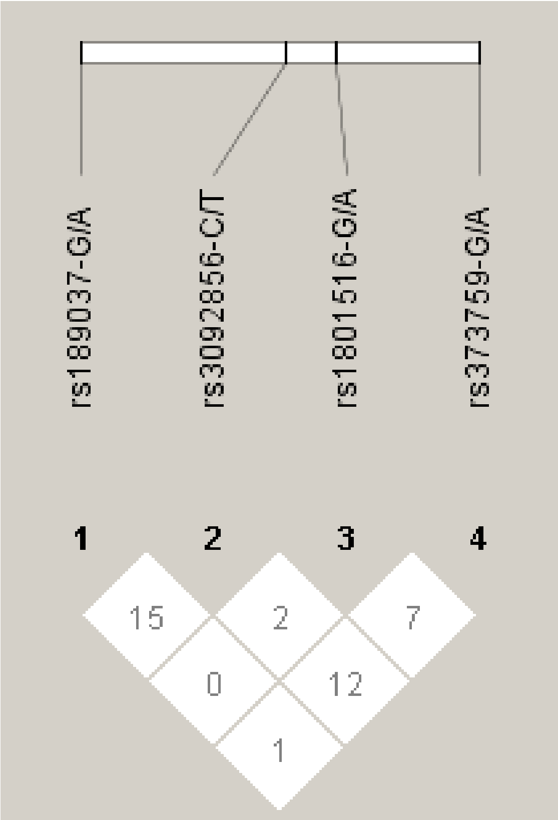

BMI-1 gene

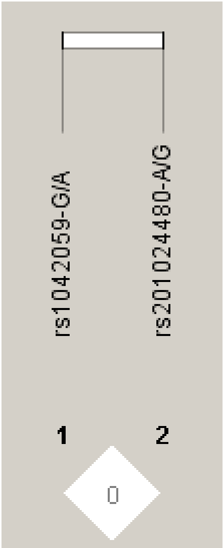

Supplementary Figure 2. Multifactor dimensionality reduction analysis of six studied variants in ATM and BMI-1 genes.

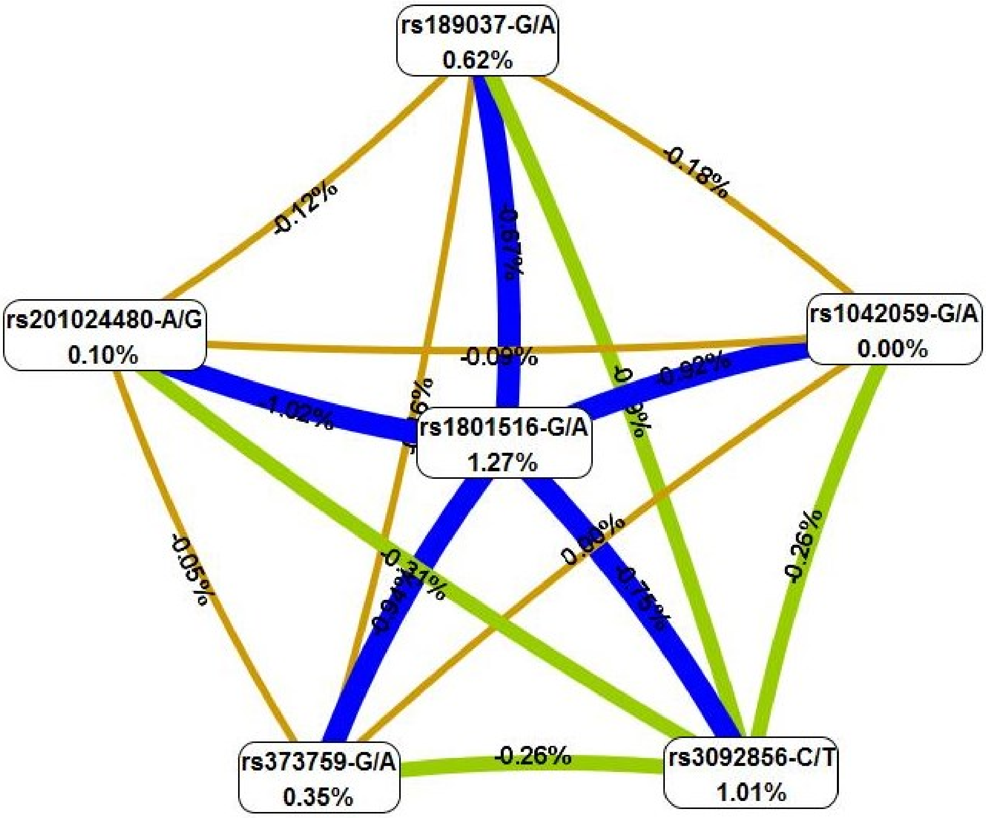

Supplement: Supplementary file 1 [file JCMM-22-3671-s001.pdf]
